# Genome-Wide Profiling of Alternative Splicing Signature Reveals Prognostic Predictor for Esophageal Carcinoma

**DOI:** 10.3389/fgene.2020.00796

**Published:** 2020-07-22

**Authors:** Jian-Rong Sun, Chen-Fan Kong, Yan-Ni Lou, Ran Yu, Xiang-Ke Qu, Li-Qun Jia

**Affiliations:** ^1^Graduate School, Beijing University of Chinese Medicine, Beijing, China; ^2^Oncology Department of Integrated Traditional Chinese and Western Medicine, China-Japan Friendship Hospital, Beijing, China; ^3^Gastroenterology Department, Beijing University of Chinese Medicine Affiliated Dongzhimen Hospital, Beijing, China; ^4^Rheumatism Department of Traditional Chinese Medicine, China-Japan Friendship Hospital, Beijing, China

**Keywords:** esophageal carcinoma, alternative splicing, survival, prognosis, splicing factor

## Abstract

**Background:**

Alternative splicing (AS) is a molecular event that drives protein diversity through the generation of multiple mRNA isoforms. Growing evidence demonstrates that dysregulation of AS is associated with tumorigenesis. However, an integrated analysis in identifying the AS biomarkers attributed to esophageal carcinoma (ESCA) is largely unexplored.

**Methods:**

AS percent-splice-in (PSI) data were obtained from the TCGA SpliceSeq database. Univariate and multivariate Cox regression analysis was successively performed to identify the overall survival (OS)-associated AS events, followed by the construction of AS predictor through different splicing patterns. Then, a nomogram that combines the final AS predictor and clinicopathological characteristics was established. Finally, a splicing regulatory network was created according to the correlation between the AS events and the splicing factors (SF).

**Results:**

We identified a total of 2389 AS events with the potential to be used as prognostic markers that are associated with the OS of ESCA patients. Based on splicing patterns, we then built eight AS predictors that are highly capable in distinguishing high- and low-risk patients, and in predicting ESCA prognosis. Notably, the area under curve (AUC) value for the exon skip (ES) prognostic predictor was shown to reach a score of 0.885, indicating that ES has the highest prediction strength in predicting ESCA prognosis. In addition, a nomogram that comprises the pathological stage and risk group was shown to be highly efficient in predicting the survival possibility of ESCA patients. Lastly, the splicing correlation network analysis revealed the opposite roles of splicing factors (SFs) in ESCA.

**Conclusion:**

In this study, the AS events may provide reliable biomarkers for the prognosis of ESCA. The splicing correlation networks could provide new insights in the identification of potential regulatory mechanisms during the ESCA development.

## Introduction

Being the seventh most frequently occurring tumor in humans, esophageal carcinoma (ESCA) ranks the sixth in causing fatalities worldwide. In year 2018 alone, the number of new ESCA cases and ESCA-related deaths was estimated to be 572,034 and 508,585, respectively (Bray et al., 2018). Although the development of early diagnosis and treatment approaches for ESCA have seen much improvement in recent years, the five-year survival rate of 15–20% is unsatisfactory (Pennathur et al., 2013). Due to the high morbidity and mortality rates of ESCA, there is an urgent call for the development of a highly efficient prognostic method. Over the past few decades, a great deal of effort has been made to identify prognostic biomarkers and therapeutic targets for ESCA. Although the studies showed some promising results, the research only focused on aspects such as mutation-driving factors and transcriptional levels (Zhu J. et al., 2018), thereby neglecting the diversity of RNA isoforms driven by post-translational modifications.

Alternative splicing (AS) is a crucial molecular mechanism by which mRNA is spliced into different RNA transcripts in order to be translated into diverse protein products (Tress et al., 2017). Recent studies showed that AS modifies about 94% of all human genes and plays an important role in the biological process (Matera and Wang, 2014; Oltean and Bates, 2014). Dysregulation of AS is associated with manifold pathological processes, including cancers where it promotes cancer development by causing the loss-of-function in tumor suppressors or the activation of oncogenes and cancer pathways. A recent study has shown multiple AS events participated in carcinogenesis, including proliferation, angiogenesis, invasion and metastasis (Mao et al., 2019). Tumor cells often tend to generate isoform switches where the variants produced are utilized to promote cell growth, drug resistance, invasion, immune escape and metastasis (Chen and Weiss, 2015; Climente-Gonzalez et al., 2017; Kim et al., 2018). For example, ZAK has two isoforms, namely ZAKα and ZAKβ (Lee et al., 2018), that play an opposite role in cancer development. Whilst ZAKα exerts an anti-neoplastic effect, ZAKβ exhibits an anti-proliferation feature. In BRCA2, one of the splicing variants BRCA2-Δ3 (Gelli et al., 2019), has been shown to be associated with a high risk of developing breast or ovarian cancer (Muller et al., 2011; Caputo et al., 2018). CXCR3 is another tumor-related gene in humans with three different splice variants: CXCR3A, CXCR3B, and CXCR3-alt. Recent studies have shown that the CXCR3 protein level is often heightened in tumor tissues than that of adjacent tissues. A high expression of CXCR3 is usually associated with adverse prognosis in cancer patients. Other studies have found that the CXCR3A variant promotes tumor cell growth while the CXCR3B variant induces tumor cell apoptosis (Ruytinx et al., 2018).

In addition, splicing factors have been shown to play a role in regulating tissue- or cell-type-specific AS (Tripathi et al., 2010), Alterations in the expression and activity of critical splicing factors can cause a string of changes to the AS, which then jointly promote tumor cell growth and survival (Ladomery, 2013). Therefore, an integrated analysis of AS events is needed in order to dissect the molecular mechanisms of ESCA and to identify potential prognostic markers for cancer.

With the continuous development of genome-wide sequencing technologies in recent years, it is now possible to identify cancer-specific molecules and prognostic biomarkers for patients (Griffith et al., 2010; Katz et al., 2010). Although systematic analysis of prognostic AS signature in liver cancer, lung cancer, head and neck cancer, and breast cancer has been reported (Suo et al., 2015; Li Y. et al., 2017; Liang et al., 2019; Wu et al., 2019), the AS signature in ESCA is largely unknown.

In the current study, we revealed numerous AS events connected with the overall survival (OS) of ESCA patients through an integrated profiling for the genome-wide AS events in the ESCA cohort from TCGA SpliceSeq. Based on the AS events identified, we constructed prognostic predictors. Then, we presented an AS-clinicopathologic nomogram which is useful in predicting the survival probability for ESCA patients. Finally, we established an SF-AS correlation network to demonstrate the underlying regulation mechanism for ESCA prognosis.

## Materials and Methods

**FIGURE 1:**
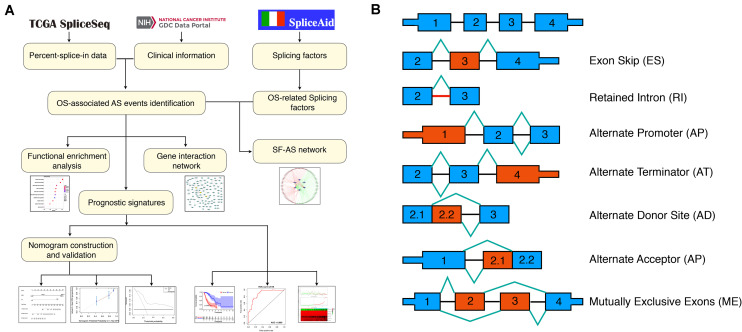
Flowchart of the present study and splicing pattern of AS events. **(A)** Study flowchart. **(B)** Illustrations for seven types of AS events, including exon skip (ES), retained intron (RI), alternate promoter (AP), alternate terminator (AT), alternate donor site (AD), alternate promoter (AP), mutually exclusive exons (ME).

The flowchart of the current study was presented in Figure 1A.

### Data Acquisition

The RNA-seq data and clinical information of the TCGA ESCA cohort were obtained from the TCGA data portal^1^; while the Percent-splice-in (PSI) data of AS events for ESCA were obtained from the TCGA SpliceSep^2^, a data portal that provides AS profiles across 33 tumors based on the TCGA RNA-seq data. There are seven types of AS events (Figure 1B) identified to date, namely Alternate Acceptor site (AA), Alternate Terminator (AT), Mutually Exclusive Exons (ME), Retained Intron (RI), Alternate Donor site (AD), Alternate Promoter (AP), and Exon Skip (ES) (Ryan et al., 2016). PSI values ranging from zero to one were used to quantify the AS events. Thus, to obtain a reliable set of AS events, we set a strict screening filter so that the percentage of samples containing PSI values exceeds 75%.

The AS events were annotated by combining the splicing type, ID number in the SpliceSeq and the corresponding parent gene symbol. For example, in “ERBB2| 99888| ES”, ERBB2 denotes the corresponding parent gene name, 99888 represents the ID of splicing variant and ES indicates the splicing type.

### Survival Analysis of AS Events, Gene Interaction Network, Functional, and Pathway Enrichment Analysis

The clinical information of ESCA patients was downloaded from the TCGA database. Based on the median PSI values, the patients were divided into two subgroups (high- and low-PSI). Univariate Cox regression analysis was conducted to detect the association between the alternative splicing (AS) events and the overall survival (OS) of ESCA patients, with *P* < 0.05 being considered significant. UpSetR (version 1.4.0) was used to create Upset plots in order to analyze the intersections of all seven types of OS-associated AS events in ESCA (Lex et al., 2014). Subsequently, the corresponding parent genes of OS-associated AS events were selected to construct a gene interaction network using Reactome FI plugin in Cytoscape (version 3.7.1), and the key genes in the network were identified using CentiScaPe2.2 plugin in Cytoscape (version 3.7.1). Functional enrichment analysis was performed by Database for Annotation, Visualization and Integrated Discovery (DAVID) online functional annotation tool^3^ using the parent genes (Dennis et al., 2003). Gene Ontology (GO) terms and Kyoto Encyclopedia of Genes and Genomes (KEGG) pathways with *P* < 0.05 were considered statistically significant. Then, the significant pathways in KEGG and the top 10 terms in each GO category, namely containing cellular (CC), molecular function (MF), and biological process (BP) were visualized by ggplot2 package in R (version 3.3.0).

### Construction of the Prognostic Predictor for ESCA Patients

Firstly, Lasso regression analysis was performed for OS-associated AS events in each splicing type in order to screen for candidates in subsequent analysis and to avoid model over-fitting. Secondly, the screened AS events were used in multivariate Cox regression analysis to construct the prognostic predictor (McNeish, 2015). Meanwhile, considering that all seven AS types have differences in their individual mode of action that is independent from each other in post-transcriptional modification, the screened AS events in each splicing type above were consolidated to construct another prognostic predictor. Then, the risk scores were computed based on each prognostic predictor and the formula used for calculating the risk score for each patient is as follows: Riskscore = β_AS event1_ × PSI_AS event1_ + β_AS event2_ × PSI_AS event2_ + ⋯ + β_AS eventn_ × PSI_AS eventn_. The patients were divided into two subgroups (high- and low-risk) according to the median risk score in order to perform Kaplan-Meier test for estimating the predictive accuracy of each prognostic predictor. The predictive accuracy of each prognostic predictor was assessed by computing the area under the curve (AUC) value at 3 years of the Receiver operating characteristic (ROC) curve by the survival ROC package (version 1.0.3). Since fewer events occurred after 5 years (see Kaplan-Meier curves), the dynamic AUC value from 1 to 5 years was calculated by time ROC package (version 0.4) in order to obtain an optimal signature. Besides, the mutations of parent genes in final signature were analyzed using maftools package in R (version 3.10).

Finally, stratified Cox survival analysis was performed to verify the independent prognostic power of the final signature in ESCA cohort such as age, gender, pathological stage and tumor grade.

### Development and Validation of an AS-Clinicopathologic Nomogram

In order to detect whether the prognostic predictor along with all clinical variables described above was associated with the OS of ESCA patients, Univariate Cox regression analysis was performed. Subsequently, the OS-related variables were used for multivariate Cox regression analysis to screen for independent prognostic factors and to develop a nomogram model that can better predict the survival probability of patients. Subsequently, to make sure that the results obtained were reliable, the nomogram model was validated by the Bootstrap method with the resample number set as 1000. The calibration curves were used to assess the predictive ability of the nomogram and the C-statistic were calculated to evaluate the discriminative ability using Hmisc package in R (version 4.1.1). A calibration curve close to 45° is an indication of good prediction ability of the model constructed by this factor. To verify clinical application of the nomogram, the decision curve analysis (DCA) was conducted using stdca package^4^.

### Construction of Underlying SF-AS Correlation Network

Splicing factors (SFs) were retrieved from the SpliceAid 2 database (Piva et al., 2012). The mRNA expression data of SFs were obtained from the TCGA database and normalized using the trimmed mean method of *M*-values (TMM) from edgeR package in R (version 3.6.0). Univariate Cox regression analysis was performed to screen the OS-associated SFs. Then, the Spearman correlation analysis was performed between the PSI values of OS-associated AS events and the expression level of OS-associated SFs, with *P* < 0.05 being set as a cut-off value. Finally, Cytoscape (version 3.7.1) was used to generate an underlying SF-AS correlation network among the significant result of spearman correlation analysis, with the correlation coefficient greater than 0.5.

## Results

### Integrated AS Events Profiles in TCGA ESCA Cohort

**FIGURE 2:**
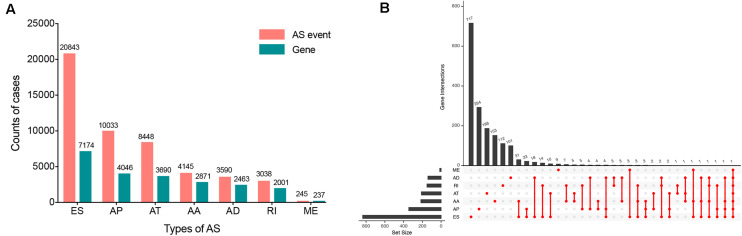
Overview of seven types of AS in this study. **(A)** Number of AS events and related genes in ESCA. **(B)** UpSet plots in ESCA, showing the interactions among the seven types of OS-associated AS events. One gene may have up to seven types of AS events.

Within the integrated AS events profiles of 185 ESCA patients from TCGA SpliceSeq, we detected a total of 50342 AS events in 10766 genes, which included 20843 ESs in 7174 genes, 10033 APs in 4046 genes, 8448 ATs in 3690 genes, 4145 AAs in 2871 genes, 3590 ADs in 2463 genes, 3038 RIs in 2001 genes, and 245 MEs in 237 genes (Figure 2A). The results showed that, among the seven types of AS events, ES was the main splicing pattern while ME was the least frequent event in ESCA patients.

### Detection and Functional Enrichment Analysis of OS-Associated AS Events

The clinical information of ESCA patients was downloaded from the TCGA database. A total of 185 ESCA patients with fully characterized tumors were included in the analysis. The demographic and clinical characteristics of patients are provided in Supplementary (Supplementary Table S1).

**FIGURE 3:**
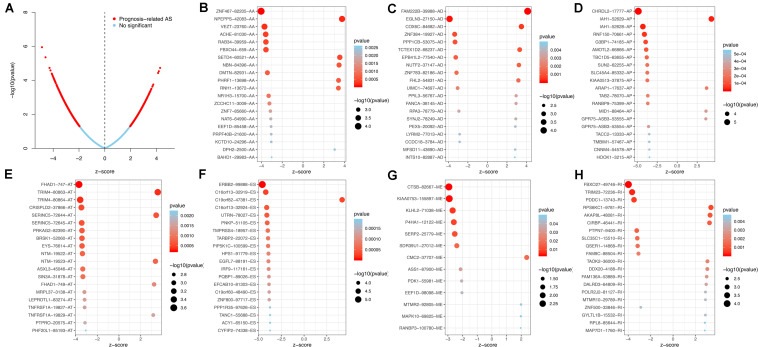
Top 20 most significant AS events in ESCA. **(A)** The volcano plots of prognosis-connected AS events. The top 20 AS events associated with survival outcome for ESCA in different splice patterns, including **(B)** AA, alternate acceptor site. **(C)** AD, alternate donor site. **(D)** AP, alternate promoter. **(E)** AT, alternate terminator. **(F)** ES, exon skip. **(G)** ME, mutually exclusive exons. **(H)** RI, retained intron.

**FIGURE 4:**
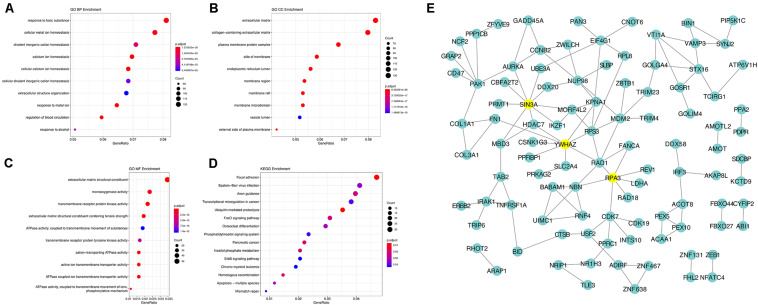
Gene interaction network and functional analysis of OS-associated alternative splicing events in ESCA. **(A)** biological processes (BP). **(B)** cellular component (CC). **(C)** molecular function (MF). **(D)** KEGG pathway analysis. **(E)** Gene interaction network of corresponding parent genes of OS-associated AS events generated by Cytoscape.

Using the AS events profiles in the ESCA cohort, we identified 2389 AS events which were significantly associated with the OS of ESCA patients (*P* < 0.05) by univariate Cox regression analysis. In particular, we found one gene with potentially more than one AS events that were significantly connected with patient survival. In order to better visualize intersecting sets, an UpSet plot was created as shown in Figure 2B. Interestingly, our analysis revealed that one gene can exhibit up to four types of AS events that were all found to be significantly associated with patient survival. Specifically, ES, AA, AD, and RI of *CIRBP* were all significantly linked to the OS of patients. The distribution of top 20 AS events in different splicing type presented in Figure 3 clearly showed that, the majority of AS event was related to good prognosis. Furthermore, all parent genes of OS-associated AS events were used in functional and pathway enrichment analysis. A total of 74 Gene Ontology (GO) terms and 15 Kyoto Encyclopedia of Genes and Genomes (KEGG) terms were identified significantly in the analysis (*P* < 0.05). The top pathways of GO and KEGG enrichment were shown in Figures 4A–D.

In order to dissect the biological relationships between the corresponding parent genes of OS-associated AS events in ESCA, a gene interaction network was created using Cytoscape. Our results revealed, three vital hub genes in the network, namely *SIN3A, YWHAZ*, and *RPA3* (Figure 4E), which may be closely related to the development of ESCA.

### Construction of the Prognostic Predictor for ESCA Patients

**FIGURE 5:**
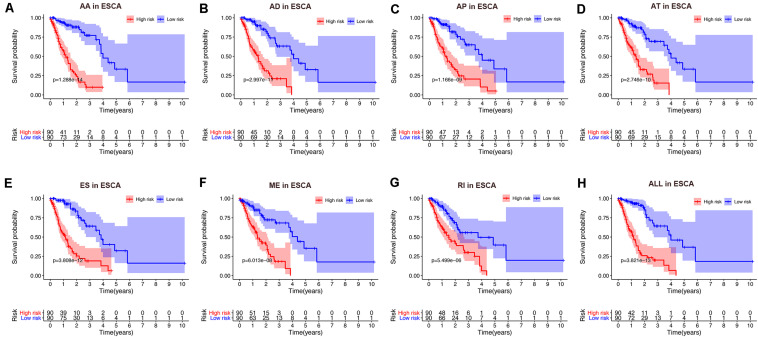
Kaplan-Meier curve of prognostic predictors constructed with either one type or all seven AS types in the ESCA cohort. **(A)** AA: alternate acceptor site. **(B)** AD: alternate donor site. **(C)** AP: alternate promoter. **(D)** AT: alternate terminator. **(E)** ES: exon skip. **(F)** ME: mutually exclusive exons. **(G)** RI: retained intron. **(H)** ALL: all seven AS types combined. Red line indicates high-risk subgroup while blue line indicates low-risk subgroup.

**FIGURE 6:**
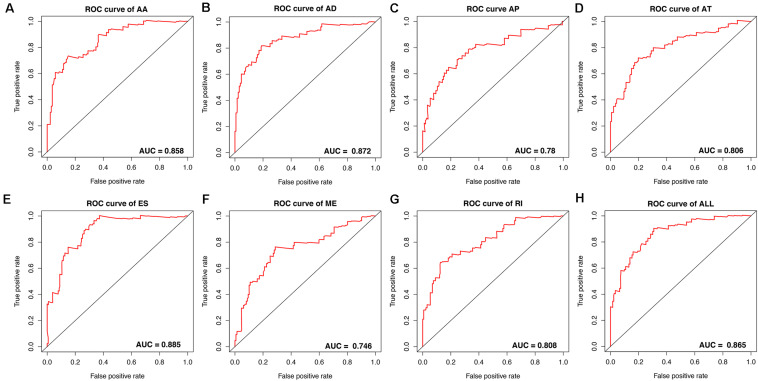
ROC curves with calculated AUC values of prognostic predictors constructed with either one type or all seven AS types in the ESCA cohort. **(A)** AA: alternate acceptor site. **(B)** AD: alternate donor site. **(C)** AP: alternate promoter. **(D)** AT: alternate terminator. **(E)** ES: exon skip. **(F)** ME: mutually exclusive exons. **(G)** RI: retained intron. **(H)** ALL: all seven AS types combined.

**FIGURE 7:**
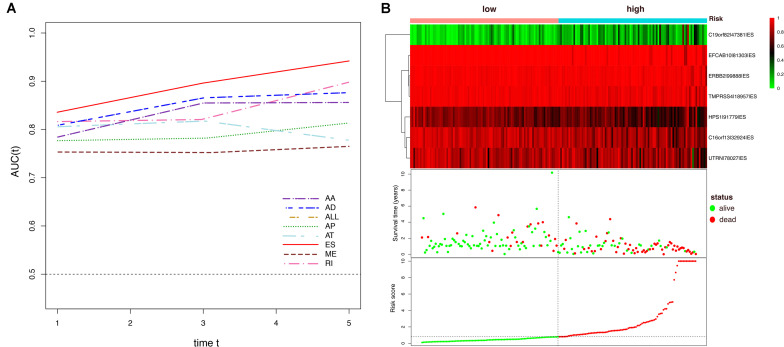
Dynamic AUC values of ROC curves for each AS model and determination of the ES prognostic signature in the ESCA cohort. **(A)** The curves of time-dependent AUCs versus time (1–5 years) of each signature: AUC(t) versus *t* **(B)** Patients were divided into high- and low-risk subgroups based on the median of risk scores based on the ES prognostic predictor. The upper part is the heatmap of AS events involved in the prognostic predictor, color transition from green to red indicates the increasing PSI score of corresponding AS event from low to high. The middle part is the survival status and survival time of each individual. Color of each plot represents the survival status of each patient. The bottom part is risk score of each individual.

**FIGURE 8:**
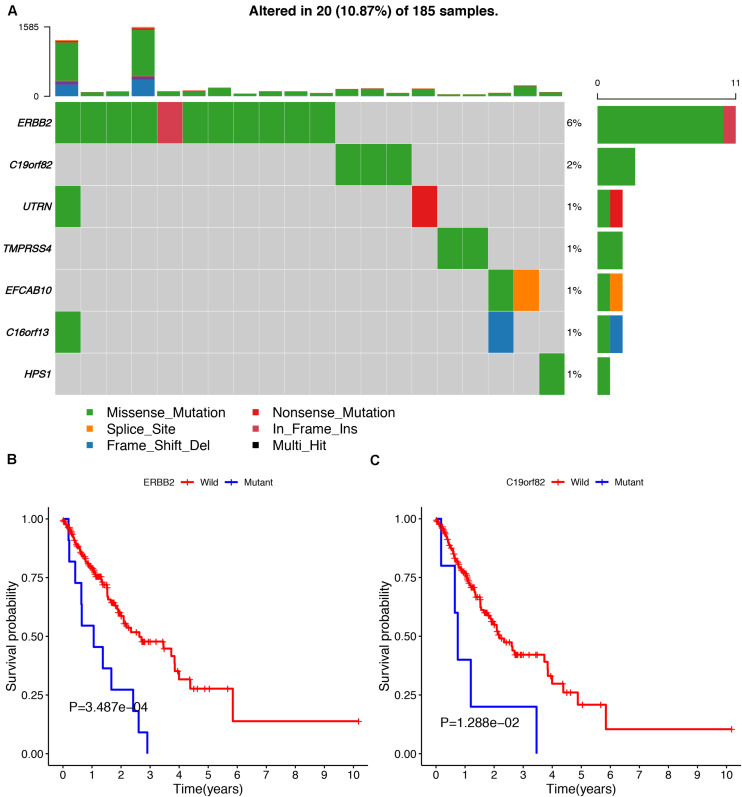
The mutation profiling of parent genes in ESCA samples. **(A)** The waterfall plot of parent genes in ESCA cohort. **(B,C)** Kaplan-Meier survival curves of two different mutated genes (ERBB2 and C19orf82).

To avoid model over-fitting, the significant OS-associated AS events (*P* < 0.05) in each AS type were analyzed by lasso regression (Supplementary Figure S1), and the results were selected to perform multivariate Cox regression analysis, respectively. Meanwhile, the AS events screened above in each splicing type were amalgamated to fit another multivariate Cox regression. Finally, a total of eight AS models were constructed, namely AA, AT, ME, RI, AD, AP, ES, and ALL models. The specific formulas of each model shown in Table 1 were used to compute the risk score of each patient, which were then divided into high- and low-risk subgroups according to the median of risk scores. Kaplan-Meier survival analysis of each model was considerably efficient in distinguishing good or poor outcome between the two subgroups (Figures 5A–H). To compare the level of efficiency among different AS models, ROC curves were created with the AUC values calculated at 3 years survival, respectively (Figures 6A–H). The AUC value of ROC for the ES prognostic predictor was calculated to be 0.885, which remained higher than other AS models over time, suggesting that ES has a higher level of efficiency than other prognostic predictors (Figure 7A). The distribution of patients’ survival status, risk score and AS events for the ES prognostic predictors as illustrated in Figure 7B showed that, the risk score increased as the patient’s survival time decreased, which resulted in a significant increase (*P* < 0.05) in the number of deaths (red dots in the upper part of Figure 7B). The corresponding parent genes of AS events included in the ES prognostic predictor were shown in Table 2. Moreover, among these seven parent genes, ERBB2 and C19orf82 possessed the most frequent genetic mutation and the missense mutation was the most common alteration (Figure 8A). The mutant of ERBB2 and C19orf82 also indicated a significantly shorter OS time than the wild type (Figures 8B,C).

**TABLE 1:** Formula of prognostic signature for esophageal carcinoma.

Type	Formula
AA	*ZNF467* |82205|AA × (−8.77) + NPEPPS|42083|AA × 3.59 + VEZT|23760|AA × (−9.88) + *RAB34* |39959|AA × (−35.9) + *FBXO44* |659|AA × (−4.92) + *SETD4* |60521|AA × 1.69 + *NR1H3* |15700|AA × (−2.05) + *ZCCHC11* |3009|AA × (−6.17)

AD	*FAM222B* |39988|AD × 4.77 + *EGLN3* |27150|AD × (−11.07) + *COX6C* |84682|AD × 3.29 + *ZNF384* |19927|AD × (−5.16) + *ZNF783* |82186|AD × (−5.33) + *UIMC1* |74697|AD × (−4.84) + FANCA|38145|AD × (6.74) + *RPA3* |78779|AD × (−31.74) + *LYRM2* |77013|AD × (−5.92)

AP	*CHRDL2* |17777|AP × (−7.74) + *IAH1* |52629|AP × 8.73 + *RNF150* |70661|AP × (−8.97) + *RANBP9* |75399|AP × (−21.7) + *GPR75*−*ASB3* |53555|AP × (3.44) + *TACC2* |13333|AP × (−3.86) + *HOOK1* |3215|AP × (−13.94)

AT	*FHAD1* |747|AT × (−1.51) + *TRIM4* |80863|AT × (3.28) + *BRSK1* |52060|AT × (−8.77) + EYS|76614|AT × (−8.45) + NTM|19522|AT × (−3.88) + *MRPL37* |3138|AT × (−9.51) + *LEPROTL1* |83274|AT × (−6.95) + PTPRO|20575|AT × (−3.16)

ES	*ERBB2* |99888|ES × (−25.65) + *C19orf82* |47381|ES × 3.35 + *C16orf13* |32924|ES × (−4.68) + UTRN|78027|ES × (−2.93) + *TMPRSS4* |18957|ES × (−11.68) + *HPS1* |91779|ES × (−5.21) + *FCAB10* |81303|ES × (−10.77)

ME	CTSB|82667|ME × (−15.83) + KIAA0753|155897|ME × (−3.57) + *KLHL2* |71038|ME × (−1.82) + *P4HA1* |12122|ME × (−3.46) + *CMC2* |37707|ME × (1.91) + *EEF1D* |98098|ME × (−1.03) + *MTMR2* |92805|ME × 2.43 + *MAPK10* |69825|ME × 2.55

RI	*FBXO27* |49746|RI × (−18.33) + *TRIM23* |72236|RI × (−28.37) + *PDDC1* |13743|RI × (−20.12) + *AKAP8L* |48081|RI × 4.51 + *PTPN7* |9400|RI × (−25.60) + *SLC35C1* |15510|RI × (−14.10) + *POLR2J2* |81127|RI × (2.55)

ALL	*CHRDL2* |17777|AP × (−7.19) + *ERBB2* |99888|ES × (−34.28) + *IAH1* |52629|AP × (6.44) + *C16orf13* |32919|ES × (−3.21) + *C19orf82* |47381|ES × 2.64 + *RNF150* |70661|AP × (−8.56) + PNKP|51105|ES × (−29.80) + *ZNF467* |82205|AA × (−8.53) + *TMPRSS4* |18957|ES × (−10.62) + *HPS1* |91779|ES × (−3.26)

**TABLE 2:** Prognostic predictors for esophageal carcinoma.

Gene	AS id	Splicing type	Exons	HR	Lower95	Upper95	*P*-value	Index
ERBB2	99888	ES	22	7.24E-12	1.86E-16	2.82E-07	1.97E-06	–25.651926
C19orf82	47381	ES	2:03	28.41024	5.787874	139.4539703	3.74E-05	3.346750
C16orf13	32924	ES	2	0.009271	0.000568	0.151374336	0.00102	–4.680888
UTRN	78027	ES	67	0.053443	0.007712	0.370361388	0.003021	–2.929139
TMPRSS4	18957	ES	11	8.48E-06	6.10E-09	0.011780172	0.001563	–11.678310
HPS1	91779	ES	9	0.005465	0.000377	0.079114333	0.000133	–5.209448
EFCAB10	81303	ES	2	2.11E-05	1.71E-08	0.026086922	0.003038	–10.765213

*AS, alternative splicing; ES, Exon Skip; HR, hazard ratio.*

Furthermore, to verify the prognostic value of the final predictor, we performed Cox survival analysis in stratified ESCA cohort where the patients were classified by clinicopathological characteristics, including age, gender, tumor grade and different pathological stages, such as T stage, M stage, and N stage. The results clearly showed that the high-risk group had a worse prognosis than that of the low-risk group in almost all cohorts (Table 3). Taken together, our results showed that the final predictor can maintain its efficiency to precisely identify patients with adverse prognosis, regardless of clinical parameters.

**TABLE 3:** Analysis of the final AS signature in stratified ESCA cohorts.

Characteristics	High risk	Low risk	HR (95% CI)	*P*-value
**Age (years)**				
≤60	47	46	5.29 (2.45-11.4)	< 0.001
>60	46	46	6.74 (2.95-15.43)	< 0.001
**Gender**				
Male	83	75	5.49 (3.14-9.60)	< 0.001
Female	11	16	7.73 (0.86-69.63)	0.068
**Tumor grade**				
G1/2	43	49	7.53 (3.09-18.35)	< 0.001
G3	31	17	9.87 (2.32-42.09)	0.002
**Pathological stage**				
Stage I/II	47	56	5.04 (2.21-11.50)	< 0.001
Stage III/IV	43	34	6.80 (3.28-14.09)	< 0.001
**T stage**				
T1/2	32	40	4.18 (1.83-9.56)	< 0.001
T3/4	56	50	6.39 (3.13-13.08)	< 0.001
**M stage**				
M0	70	75	5.57 (2.99-10.37)	< 0.001
M1	8	4	7.32 (0.88-60.59)	0.065
**N stage**				
N0	34	42	6.63 (2.10-20.93)	0.001
N1	37	34	4.84 (2.41-9.73)	< 0.001
N2/3	18	11	5.07 (1.45-17.72)	0.011

*HR, hazard ratio; CI, confidence interval.*

### Development and Efficiency of AS-Clinicopathologic Nomogram

**FIGURE 9:**
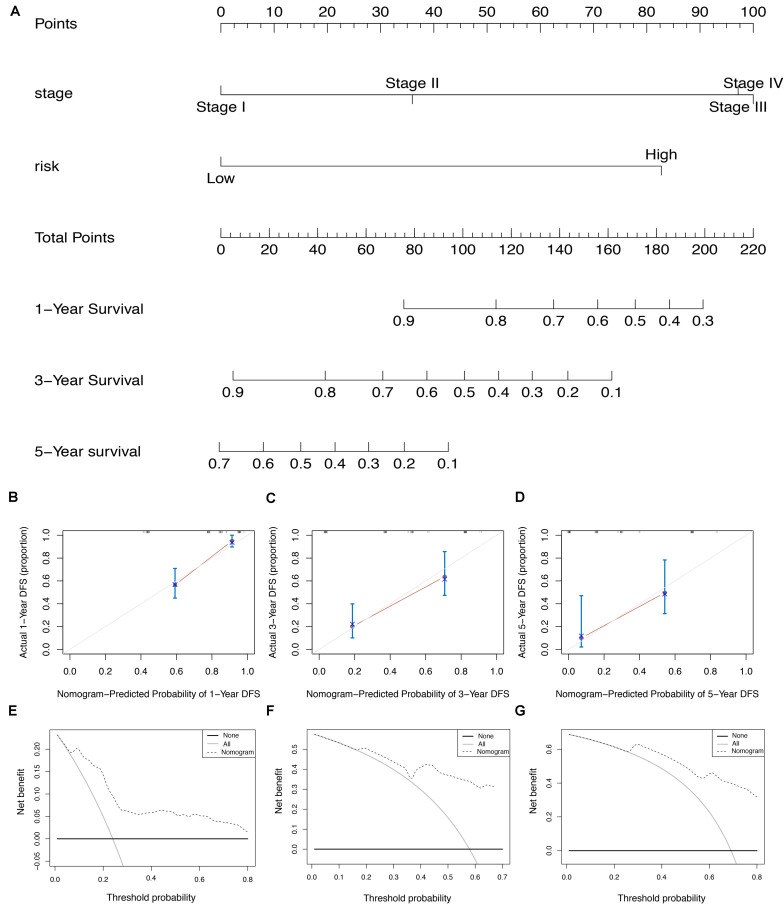
The AS-clinicopathologic nomogram for prediction on survival probability in patients with ESCA. **(A)** Development of AS-clinicopathologic nomogram for predicting 1-, 3-, and 5-years OS for ESCA patients. **(B–D)** Calibration plot of the AS-clinicopathologic nomogram in terms of agreement between nomogram-predicted and observed 1-, 3-, and 5-years outcomes in the ESCA cohort. The actual performances of our model are shown in red lines. And the silver line of 45° represents the ideal performance. **(E–G)** Decision curve analyses of the AS-clinicopathologic nomogram for 1-, 3-, and 5-years risk in ESCA cohort. The gray line represents the net benefit of treat-all scheme varying with threshold probability, while the black line represents the net benefit of treat-no scheme. The net benefits by using our nomogram for predicting 1-, 3-, and 5-years OS are displayed with imaginary line.

To screen for potential factors correlated with the OS of ESCA patients, the risk level (high or low) based on the ES prognostic predictor along with clinicopathologic variables mentioned earlier were studied by univariate Cox analysis. The results showed that tumor grade, pathological stage and risk score level were statistically significant (*P* < 0.05) (Table 4). Multivariate Cox regression analysis revealed that the risk score level derived from the ES prognostic predictor and the pathological stage were the only independent prognostic factors associated with the OS of ESCA patients (Table 4). These independent prognostic factors were used in the construction of subsequent nomograms (Figure 9A). The calibration curve of the nomogram for the probability of survival at 1, 3, 5 years showed good uniformity between prediction and actual observation (Figures 9B–D). The C-statistic for OS prediction of ESCA patients was 0.78, indicating that the predictive ability of this nomogram model was efficient. The DCA of this nomogram for 1, 3, 5 years as shown in Figures 9E–G demonstrated that this nomogram had good clinical usefulness, which meant that if the threshold probability was less than 80%, using this nomogram to predict prognosis in 1, 3, or 5 years added more benefit than either the treat-none scheme or treat-all scheme.

**TABLE 4:** Univariate and multivariate Cox regression analysis for clinical variables.

Variables	Univariate analysis		Multivariate analysis	
	HR (95% CI)	*P-*value	HR (95% CI)	*P-*value
Age	1.01 (0.99–1.03)	0.45	−	−
Gender	2.92 (0.91–9.38)	0.07	−	−
Tumor grade	1.63 (1.07–2.48)	0.02	1.15 (0.66–1.98)	0.63
Pathological stage	2.51 (1.74–3.61)	< 0.001	3.03 (1.12–8.18)	0.03
T	1.65 (1.13–2.41)	< 0.01	1.05 (0.56–1.62)	0.86
N	1.76 (1.33–2.34)	< 0.001	1.03 (0.60–1.75)	0.93
M	2.93 (1.30–6.58)	< 0.01	1.25 (0.08–2.55)	0.36
Risk score	1.17 (1.12–1.22)	< 0.001	1.13 (1.08–1.19)	< 0.001

*The “−” indicates that the value is not available; HR, hazard ratio; CI, confidence interval.*

### Establishment of the SF-AS Correlation Network

**FIGURE 10:**
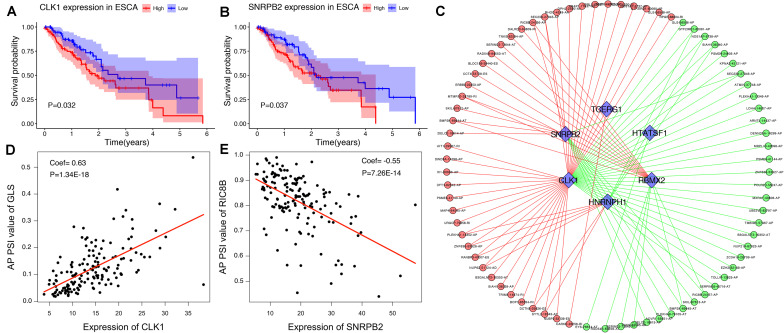
The OS-associated splicing factors in ESCA and SF-AS correlation network. **(A,B)** Kaplan-Meier curves of survival-related splicing factors. **(C)** SF-AS correlation network. Blue triangles were OS-associated splicing factors. Red/green lines represent positive/negative correlations between substances. Red/green dots represent adverse/favorable AS events. **(D,E)** Dot plots of correlations between expression of SFs (CLK1 and SNRPB2) and PSI values of OS-associated AS events.

To explore the upstream mechanism of AS regulation, we calculated the gene expression levels of SFs from the TCGA ESCA level 3 RNA-seq data and subsequently conducted univariate Cox regression analysis. The results showed that a total of 15 SFs were significantly related to the OS of ESCA patients (*P* < 0.05) (Supplementary Table S2). For instance, the expression level of SFs *CLK1* and *SNRPB2* was found to be associated with poor prognosis (Figures 10A,B). In addition, the correlations between the PSI values of OS-associated AS events and the gene expression levels of OS-associated SFs were investigated using Spearman’s test. Our analysis identified a total of six key SFs that are associated with poor prognosis, including *CLK1, SNRPB2, TCERG1, HTATSF1, RBMX2*, and *HNRNPH1*, indicating that the abnormal expression of these key SFs may play a role in the dysregulation of the splicing patterns in ESCA. The correlation network as shown in Figure 10C revealed a total of 5 OS-associated SFs (blue triangles) that were significantly correlated with 77 OS-associated AS events (red and blue dots). The red dots indicate adverse prognosis (HR > 1) while green dots denote favorable clinical outcomes (HR < 1). Additionally, we found that most adverse survival prognostic AS events (red dots) were positively correlated (red lines) with the expression of SFs (blue triangles); while most favorable prognosis AS events (green dots) were negatively correlated (green lines) with the expression of SFs. The representative dot plots of correlation between the SFs and AS events were shown in Figures 10D,E. Based on our observations, we bypothesize that the oncogenic SFs play a key role in meditating the dysregulation of AS in ESCA, which leads to cancer development.

## Discussion

AS is a post-translational modification process that generates multiple mRNA isoforms from a single gene. The resulting RNA transcripts can function differently and participate in various physiological processes. Dysregulation of AS in cancer-related genes has been found to participate in many biological processes in tumors, and these abnormally regulated genes can be used as molecular markers for cancer prognosis and treatment. However, an integrated analysis of the AS signature in ESCA remains largely unknown.

In this study, we performed a systematic analysis of OS-associated AS events in 185 of ESCA patients from TCGA SpliceSeq. A total of 2389 AS events were found to be significantly associated with the OS of ESCA patients. Among these OS-associated AS events, some splice variants that have been identified to play an important part in tumor biology were also included in our analysis. For instance, ECM1b, a splice isoform derived from ECM1 (due to an ES event based on our data) can enhance chemosensitivity by suppressing MTORC2/MYC/MTORC1 signaling pathway. One study has demonstrated that ECM1b expression sensitizes ESCA cells to cisplatin, a drug commonly used in ESCA patient treatment (Yu et al., 2019). MUC1, a spliced variant of PUF60 (following an ES event based on our data) can promote carcinogenesis by regulating P53 and β-catenin. An increased expression level of MUC1 is associated with malignant transformation of various malignancies in different tissues, such as breast, colon and pancreas. MUC1 itself has nine main splice variants in which MUC1/C, D and Z are associated with cancer progression (Kahkhaie et al., 2014). Therefore, our comprehensive analysis of AS events nicely complements the AS atlas of ESCA.

The carcinogenesis of ESCA is correlated to multiple pathological processes with a complicated regulatory network. Therefore, predicting tumor prognosis by amalgamating multiple biomarkers and establishing a model is far more effective than that of using a single clinical indicator. Over the past decade, numerous studies have integrated genome-wide prognostic biomarkers to improve the prognosis and diagnosis of ESCA. However, most studies are limited at the transcriptome level, as the focus were given to mRNA, lncRNA or miRNA as the prognostic predictors (Fan and Liu, 2016; Xue et al., 2018). In this study, we focused on AS which belongs to the gene posttranscriptional regulation level. Therefore, we created the prognostic predictors for each type of AS by multivariate Cox regression analysis. Our results showed that the ES model with the best AUC value at 0.885 exhibited a high prediction efficiency than other models. Some parent genes of AS events in the ES model have also been reported to play critical roles in cancer biology. For instance, TMPRSS4, a type-II transmembrane serine protease found to be upregulated in many solid cancers can promote the proliferation, invasion and migration of cancer cells (Jin et al., 2016; Li X.M. et al., 2017; Jianwei et al., 2018). ERBB2, a common oncogene that has been used as one of the key prognostic and treatment indicators in breast cancer, exhibits an overexpressed level in approximately 25–30% of breast cancers and confers a worse biological effect. Besides breast cancer, ERBB2 overexpression is also commonly detected in gastric, esophageal and endometrial cancers (Moasser, 2007). Notably, ES was found to be the most frequent splicing type in our study. In agreement with this, some studies have shown that some splicing variants of genes generated through ES was upregulated in some solid cancers, and can increase the motility of cancer cells (Oltean and Bates, 2014). D16ERBB2, a splice variant of ERBB2 generated through the skipping of exon 16, has been shown to exert high tumorigenecity, and a close association with increased tumor invasive properties and metastasis (Gautrey et al., 2015). Interestingly, our analysis showed that the AS events of ERBB2 is a favorable prognostic predictor, indicating that depending on the exon deletion site, the resulting splicing variant may play an entirely opposite role in tumor development. However, few studies have reported the detailed biological significance of other parent genes in the ES model. Hence, the underlying mechanism of these splicing events involved in final model is largely unclear. Therefore, further research with functional experiments is urgently in need.

Furthermore, to enable the prognostic predictor achieve a more reliable and valuable prediction efficacy in clinical settings, the prognostic nomogram that comprises the pathological stage and the risk level based on the ES prognostic predictor, was developed for assessing individual survival risk of patients with satisfactory discrimination. The calibration curve, C-statistic, and DCA curve demonstrated that the nomogram had great potential to be applied in clinical practice. Moreover, we performed functional enrichment analysis to explore the biological function of AS events in ESCA. Our CC of GO enrichment analysis showed that AS can mediate extracellular matrix-related pathways to promote tumor cell proliferation, invasion and metastasis (Wang et al., 2016). Additionally, KEGG analysis revealed several significant signaling pathways, such as ubiquitin-mediated proteolysis and focal adhesion signaling, which were consistent with the comprehensive analysis of AS in gastrointestinal adenocarcinomas and correlated with the tumorigenesis and prognosis of ESCA (Lin et al., 2018; Zhu R. et al., 2018). Therefore, we hypothesize that the cancer-associated outcomes due to AS alteration may be associated with these common pathways.

As the main regulator of the AS event, SF can affect the choice of splicing sites through recognition and binding of the mRNA precursor. In this study, we identified 6 SFs (*CLK1, SNRPB2, TCERG1, HTATSF1, RBMX2*, and *HNRNPH1*) associated with adverse prognosis of ESCA. Some of these SFs have been reported previously. For example, HNRNPH1, an RNA-binding protein highly expressed in many cancers, was found to alter the splicing of some oncogenes following knockdown, which then inhibits the tumor formation and growth in Rhabdomyosarcoma (Li et al., 2018). CLK1, a member of the CLKs family that phosphorylates SR proteins involved in splicing, was shown to promote the phosphorylation of SPF45 when overexpressed, which ultimately induces cell migration and invasion of ovarian cancer (Liu et al., 2013). Finally, our SF-AS correlation network outlined an obvious trend, showing that whilst most favorable prognostic AS events were negatively associated with the expression level of SFs in ESCA; adverse prognostic AS events were positively associated with the expression level of SFs. Notably, this phenomenon proposed an assumption that the dysregulation of AS in ESCA was related to the up-regulation of SFs. This study provided another approach to understand the splicing patterns and their mechanistic connection to SFs in the ESCA, which will enable us to dissect the potential mechanism of AS events in the development of ESCA.

Although our predictor performed well in ESCA prognosis prediction, there are inevitably several limitations in the current study that can be improved. Firstly, the number of patients included in the ESCA cohorts were limited. Secondly, this study lacks other independent cohort of ESCA patients that can be used to demonstrate the reproducibility of the prognostic predictors constructed in this report. Nevertheless, our comprehensive analysis of the splicing pattern provides some fundamental knowledge to study the molecular mechanism and to identify potential drug targets for ESCA.

## Conclusion

In conclusion, we performed an integrated analysis for RNA splicing patterns of ESCA and constructed a prognostic predictor that can be used to predict the survival probability of ESCA patients. More importantly, we constructed a well-executed nomogram that combines clinicopathological variables with the final prognostic predictor, which showed a great potential to be applied in clinical settings. The correlation network between prognostic AS events and SFs suggested a potential mechanism of the oncogenic process in ESCA. Additionally, the AS events revealed in our study, particularly those that can be used as a prognostic predictor, exhibited considerable potential for clinical application as prognostic markers as well as therapeutic targets. Our study also provided valuable fundamental knowledge to understand the underlying mechanism of ESCA development.

## Data Availability Statement

The datasets generated for this study can be found in the TCGA SpliceSeq (https://bioinformatics.mdanderson.org/TCGASpliceSeq/), the TCGA database (https://portal.gdc.cancer.gov, version 18.0).

## Ethics Statement

As our data were downloaded from the TCGA SpliceSeq and TCGA database, there is no requirement for ethics committee approval and consent to participate.

## Author Contributions

L-QJ contributed to design the study and revised the manuscript. J-RS, C-FK, Y-NL, X-KQ, and RY collected and assembled the data. J-RS, C-FK, and Y-NL conducted the data analysis and interpretation. J-RS drafted the manuscript. All the authors read and approved the final manuscript.

## Conflict of Interest

The authors declare that the research was conducted in the absence of any commercial or financial relationships that could be construed as a potential conflict of interest.

## Footnotes

1 https://portal.gdc.cancer.gov, version 18.0

2 https://bioinformatics.mdanderson.org/TCGASpliceSeq/

3 https://david.ncifcrf.gov/, version 6.8

4 https://www.mskcc.org/

## Supplementary Material

FIGURE S1 Lasso regression analysis for different types of OS-associated AS events. **(A)** AA: alternate acceptor site. **(B)** AD: alternate donor site. **(C)** AP: alternate promoter. **(D)** AT: alternate terminator. **(E)** ES: exon skip. **(F)** ME: mutually exclusive exons. **(G)** RI: retained intron. **(H)** ALL: all types of AS events.

TABLE S1 Clinical parameters of patients from the TCGA.

TABLE S2 The survival-associated splicing factors.

## Abbreviations

AA: alternate acceptor site
AD: alternate donor site
AP: alternate promoter
AS: alternative splicing
AT: alternate terminator
AUC: area under curve
DCA: decision curve analysis
ES: exon skip
ESCA: esophageal carcinoma
HR: hazard ratio
ME: mutually exclusive exons
OS: overall survival
PSI: Percent Spliced In
RI: retained intron
RNA-seq: RNA sequencing
ROC: receiver operating characteristic
SFs: splicing factors
TCGA: The Cancer Genome Atlas.
