# Supplementary material for: Genome-Wide Profiling of Alternative Splicing Signature Reveals Prognostic Predictor for Esophageal Carcinoma
Source: Front Genet. 2020 Jul 22;11:796. doi: 10.3389/fgene.2020.00796 (PMC7387693; doi:10.3389/fgene.2020.00796)
Supplement: TABLE S1 — Clinical parameters of patients from the TCGA. [file Table_1.DOCX]

| Supplementary Table 1. Clinical parameters of patients from the TCGA | |
| --- | --- |
| **Parameter/Feature** | **ESCA  (N=185)** |
| Age, years (mean±SD) | 62.45±11.89 |
| Gender, male | 158 |
| Tumor grade |  |
| G1 | 19 |
| G2 | 77 |
| G3 | 49 |
| GX | 40 |
| Pathologic stage |  |
| I | 21 |
| II | 86 |
| III | 66 |
| IV | 12 |
| Tumor size |  |
| T0 | 2 |
| T1 | 32 |
| T2 | 44 |
| T3 | 102 |
| T4 | 5 |
| Lymph node |  |
| N0 | 80 |
| N1 | 74 |
| N2 | 21 |
| N3 | 8 |
| NX | 2 |
| Metastasis status |  |
| M0 | 150 |
| M1 | 12 |
| MX | 23 |
