# Supplementary material for: Genome-Wide Profiling of Alternative Splicing Signature Reveals Prognostic Predictor for Esophageal Carcinoma
Source: Front Genet. 2020 Jul 22;11:796. doi: 10.3389/fgene.2020.00796 (PMC7387693; doi:10.3389/fgene.2020.00796)
Supplement: TABLE S2 — The survival-associated splicing factors. [file Table_2.DOCX]

| Supplementary Table 2: The survival-associated splicing factors | | | | |
| --- | --- | --- | --- | --- |
| **Gene** | **HR** | **Lower95** | **Upper95** | ***P*-value** |
| SNRPB2 | 1.035 | 1.013 | 1.058 | 0.002 |
| FAM50A | 1.048 | 1.014 | 1.082 | 0.005 |
| HSPA1B | 1.008 | 1.002 | 1.014 | 0.008 |
| ZCCHC10 | 1.225 | 1.048 | 1.433 | 0.011 |
| CLK1 | 1.047 | 1.008 | 1.088 | 0.018 |
| HTATSF1 | 1.030 | 1.004 | 1.057 | 0.023 |
| ZMAT5 | 0.663 | 0.464 | 0.949 | 0.025 |
| HSPA1A | 1.005 | 1.000 | 1.010 | 0.035 |
| RNF40 | 0.915 | 0.841 | 0.995 | 0.038 |
| RBMX2 | 1.063 | 1.003 | 1.127 | 0.041 |
| DDX46 | 1.114 | 1.004 | 1.235 | 0.042 |
| ZC3HAV1 | 1.063 | 1.002 | 1.127 | 0.042 |
| TCERG1 | 1.099 | 1.001 | 1.207 | 0.047 |
| CIRBP | 0.945 | 0.893 | 1.000 | 0.048 |
| HNRNPH1 | 1.026 | 1.012 | 1.045 | 0.006 |
